# Leukopenia and agranulocytosis in atypical antipsychotic treatment - besides clozapine

**DOI:** 10.1192/j.eurpsy.2021.2112

**Published:** 2021-08-13

**Authors:** B. Jorge, C. Pedro Fernandes, J. Carvalho

**Affiliations:** Serviço De Psiquiatria, Hospital de Braga, Braga, Portugal

**Keywords:** Antipsychotics, Haematological effects, Leukopenia/agranulocytosis

## Abstract

**Introduction:**

Leukopenia and agranulocytosis are reported and dangerous haematological side-effects associated with the use of antipsychotics, mostly reported for clozapine administration. However, increased case reports about severe abnormalities even during treatment with second generation antipsychotics other than clozapine.

**Objectives:**

This review aims to compare haematological abnormalities associated with clozapine vs non-clozapine antipsychotic treatment, regarding aspects such as safety levels or the need for regular blood samples monitoring.

**Methods:**

Pubmed and Google Scholar were searched for eligible articles, through keyword search and cross-referencing.

**Results:**

Neutropenia is common both in patients with schizophrenia on clozapine treatment and in those never on clozapine. Cases of agranulocytosis has been described with the use of olanzapine, risperidone or paliperidone, that do not have the same monitoring regulatory process as clozapine.

**Conclusions:**

These results highlight the challenges in identifying and managing non-clozapine antipsychotic-induced leukopenia in susceptible patients. Continued research in this domain for evidence based management of antipsychotic-induced blood dyscrasias

**Disclosure:**

No significant relationships.

